# *Tropaeolum* Tops Tobacco – Simple and Efficient Transgene Expression in the Order Brassicales

**DOI:** 10.1371/journal.pone.0073355

**Published:** 2013-09-10

**Authors:** Andrea Pitzschke

**Affiliations:** Department of Applied Genetics and Cell Biology, University of Natural Resources and Applied Life Sciences, Vienna, Austria; Key Laboratory of Horticultural Plant Biology (MOE), China

## Abstract

Transient expression systems are valuable tools in molecular biology. Agrobacterial infiltration of leaves is well-established in tobacco, but has led to limited success in the model plant *Arabidopsis thaliana*. An efficient expression system combining the advantages of *Arabidopsis* (well-characterised) and the simplicity of leaf infiltration is desirable. Here, I describe *Agrobacterium tumefaciens*-mediated transformation of *Tropaeolum* *majus* (nasturtium, order Brassicales) as a remarkably simple, cheap and highly efficient transient expression system. It provides the *Arabidopsis* community with a tool to study subcellular localisation, protein–protein interactions and reporter gene activities (e.g. luciferase, β-glucuronidase) in a genetic background that is closely related to their primary model organism. Unlike *Arabidopsis* *,* *Tropaeolum* is capable of engaging in endomycorrhizal associations and is therefore relevant also to symbiosis research. RNAi-based approaches are more likely to succeed than in the distantly-related *Nicotiana* transformation system. *Tropaeolum* *majus* was voted the “medicinal plant of the year 2013”. Conquering this plant for genetic manipulations harbours potential for biotechnological and pharmacological applications.

## Introduction

The soil-born *Agrobacterium tumefaciens* is the only organism capable of interkingdom gene transfer. It has been employed intensively for genetic manipulation of plant cells. Transformation is accomplished through the action of both bacterial and host proteins, many of which have been identified and functionally characterised [1]. Yet, it is still impossible to predict which plant species are easily accessible and which are recalcitrant to *Agrobacterium* -mediated transformation.

*Arabidopsis thaliana* is the best-characterised plant, whose properties (small and fully sequenced genome, short regeneration time, modest growth requirements) have facilitated research fundamentally [2,3]. Its close relatedness to several agriculturally relevant plants, such as rapes, cabbage and mustard, contribute to the popularity and relevance of *Arabidopsis* as model organism in plant science. In *Arabidopsis* , *Agrobacterium* -mediated T-DNA transfer is primarily employed to generate stable transgenic plants. Transient transformation efficiency is low, it strictly depends on growth conditions and has yielded satisfactory results only in young seedlings [4]. In contrast, stable transformation is conveniently achieved through the “floral dip” method [5], in which plants at the flowering stage are submerged into a suspension of agrobacteria carrying the desired transformation plasmid. Primary transformants are obtained within few months. However, if one wishes to transfer multiple transgenes, different antibiotic markers are needed to secure reliable selection. Alternatively, characterised transgenic individuals can be crossed, and individuals carrying both transgenes identified by genotyping. Although these approaches have their merits, substantial time and space are needed. For more rapid answers as to the subcellular localisation of a protein of interest, trans-activation capacity of transcription factors or protein–protein interactions transient expression systems are the method of choice. The most commonly used approaches are bombardment, PEG-mediated protoplast transformation and *Agrobacterium* -mediated transformation [6,7]. Transgenes can be introduced into protoplasts obtained from suspension cultures or from freshly isolated leaf mesophyll cells. The recently reported tape- *Arabidopsis* -sandwich strategy offers a further simplification of this technique [8]. Similarly easy transformation, allowing transfer of multiple transgenes at a time, has been achieved in Poinsettia [9]. However, only comparatively few species are amenable to protoplast transformation, and optimisation of isolation/transformation protocols are time-consuming. In addition, data obtained from studies in protoplasts, which are deprived of their cell wall and tissue context, need to be interpreted with caution.

**Figure 1:**
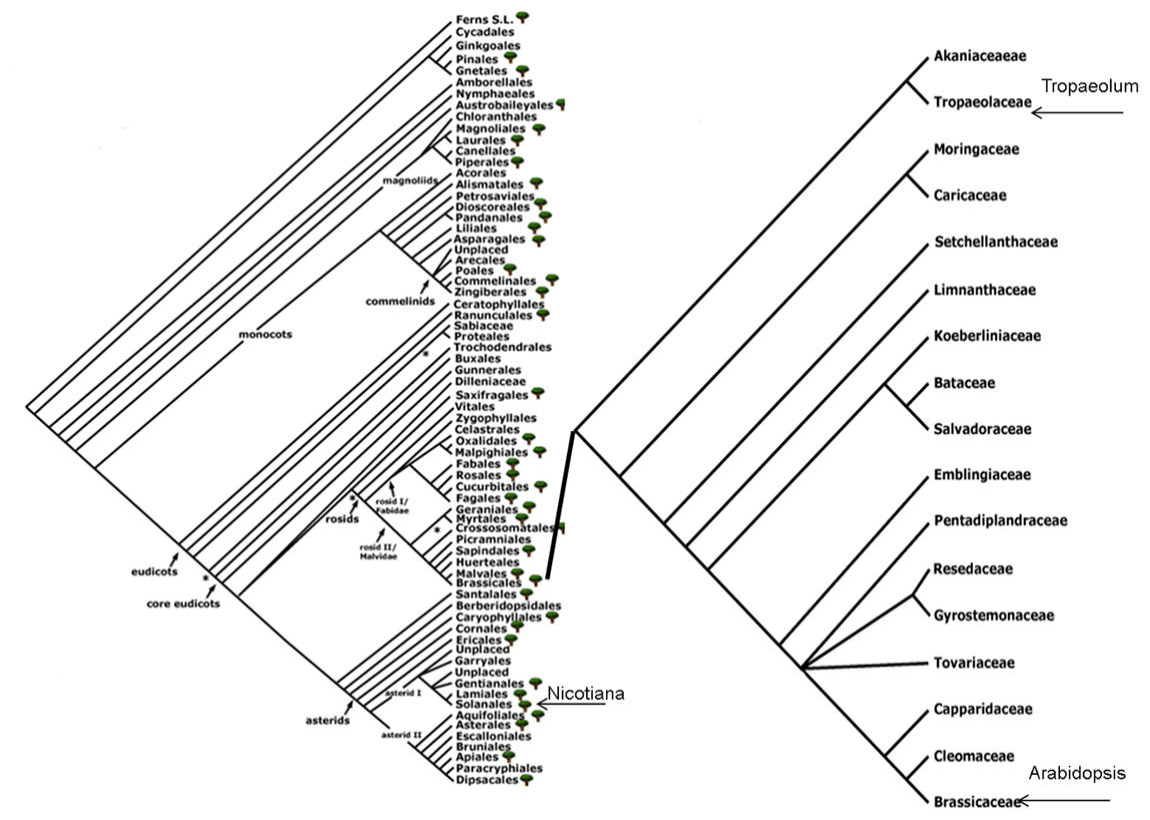
Angiosperm phylogeny and phylogenetic relation within the order Brassicacales. Positions of plant species mentioned in the manuscript are indicated. The image was modified from the „Angiosperm Phylogeny Website – Missouri Botanical Garden“. Reprinted from http://www.mobot.org/MOBOT/research/APweb/ under a CC BY license, with permission from Peter F. Stevens, original copyright 2013. (http://www.mobot.org/MOBOT/research/APweb/).

An alternative approach for multiple transgene expression is *Agrobacterium* -mediated transformation. Again, this method has its merits and limitations. It does allow analysis in intact tissue, is easy to perform and a low-cost method. On the other hand, by definition, it strictly relies on the presence of the microbial pathogen. Agrobacteria carrying the construct(s) of interest are infiltrated into leaves of intact plants, and transgene expression can be studied within 3-14 days. *Nicotiana tabacum* [10] and *Nicotiana* *benthamiana* [11] are the favoured species for this approach. They are easy to propagate, and agrobacterial infiltration is normally performed in 6-8 week-old plants. For regular supply and growing of plants significant space is required. Also, in general a maximum of 4-6 leaves can be infiltrated per plant. Within a given plant, transformation efficiencies can differ substantially depending on the type of leaf (upper, lower) and leaf area (margin; distance to petiole etc.) being infiltrated [12]. Plants tend to flower early (<10 weeks) under the conditions optimised for their regular supply; and are normally not used at this stage anymore. However, most importantly, for the study of *Arabidopsis* -derived transgenes, *Nicotiana* is a sub-optimal system, since it is only distantly related to *Arabidopsis* .

The motivation of this study was to identify a plant species that is a) related to *Arabidopsis* , b) modest in its growth requirements (light/humidity), c) less space-occupying than *Nicotiana* , d) easy to infiltrate with *Agrobacteria* and e) transformable at high efficiency. *Tropaeolum* *majus* (nasturtium) fulfils all above requirements.

Here, I describe *Agrobacterium* -mediated transformation of *T* *. majus* as a convenient, cheap and efficient transient expression system. It facilitates studies in a genetic background that is closely related to the model plant *Arabidopsis* . In addition, it offers an alternative and complementary method to *Nicotiana* leaf infiltration. The accessibility of *Tropaeolum* to simple and fast genetic manipulation potentially drives progress in several fields of plant research, including those aimed at biotechnological and pharmacological applications. Unlike *Arabidopsis* *,* *Tropaeolum* is capable of engaging in endomycorrhizal associations, and is therefore also of interest to symbiosis researchers. Besides, it was voted the “medicinal plant of the year 2013”, and the here-described properties may contribute to its “fame” and popularity in the scientific community.

## Materials and Methods

### Plant growth and transformation

*Tropaeolum* *majus* L. seeds were directly placed into pots and covered with 1 cm soil. Pots were kept moist and placed at room temperature (20-24 °C, no special growth chamber, range of air humidity/temperature/light intensity tested). Plants that had formed at least 4-6 leaves were used for infiltration. *Agrobacterium tumefaciens* (GV3101) carrying the Ti helper plasmid pSOUP and a pGreen-derived [13] construct of interest were streaked from glycerol cultures onto a LB agar plate supplemented with rifampicin, tetracycline and kanamycin. After 2 days at 28 °C, loops of bacterial cells were transferred into 1.5 ml reaction tubes and resuspended in 1 ml infiltration liquid (IF): 10 mM MES, pH5.7; 10 mM MgCl2 and 100 µM acetosyringone. Samples were centrifuged, the supernatant fluid discarded, and the bacterial pellet was resuspended in IF, adjusted to an OD (600 nm) of 0.2. The bacterial suspensions were incubated at least 3 hours (dark, RT, no shaking) before infiltration.

### Plasmids

pGreen derivatives carrying the CaMV35S promoter and transgenes for YFP, MKK4-SPYCE, MPK3-SPYNE or GUS have been described previously [14,15]. The coding region of luciferase was isolated as NcoI-XbaI fragment from pGL3 (Promega) and used to replace a YFP-encoding fragment in plasmid CaMV35S::YFP. *Arabidopsis* gene accession numbers are: MPK3: At3g45640, and MKK4: At1g51660.

**Figure 2:**
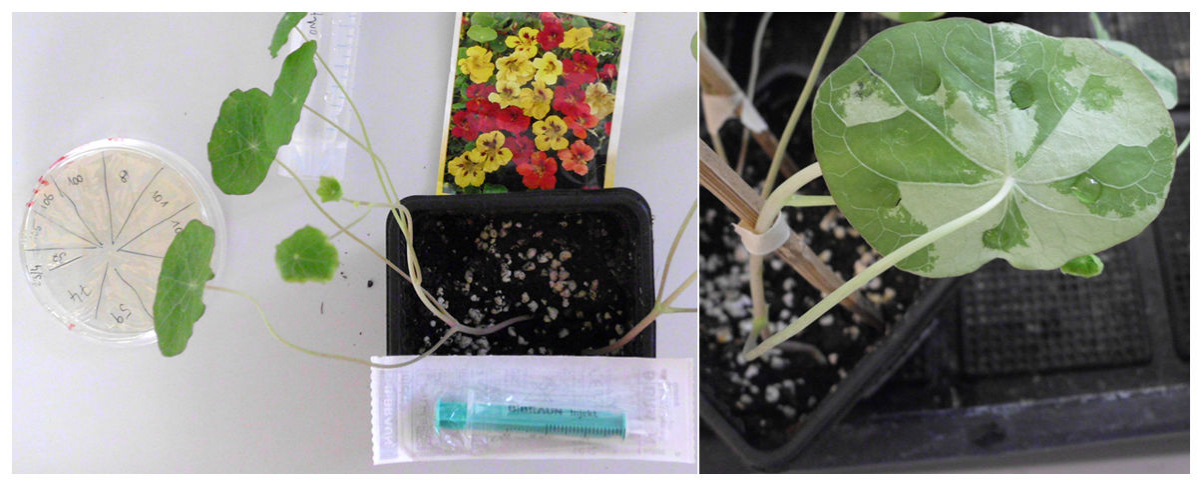
Outline of the *Tropaeolum* transformation procedure. Seeds are sown on soil and grown until at least four leaves have developed (app. 2 weeks). Agrobacteria carrying the construct of interest are streaked from glycerol cultures onto LB (incl. Antibiotics) plates for 2 days. Loops of cells are briefly washed and resuspended in infiltration liquid, adjusted to OD600nM 0.2, incubated for 3 hours and subsequently infiltrated into the leaves.

### UV microscopy

YFP expression and protein–protein interactions studies were conducted at a UV microscope (Leica DM5500B), equipped with excitation/emission filters: BP450–450 nm/LP515 nm as described previously [15].

**Figure 3:**
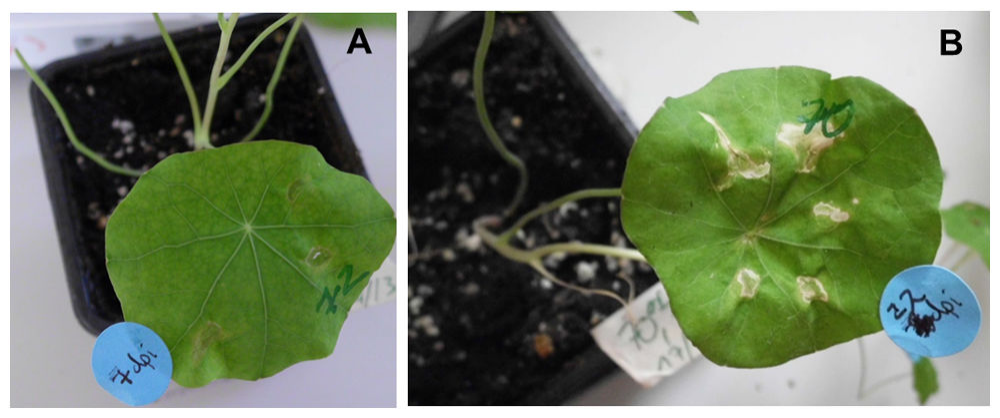
Leaf appearance after infiltration. **A**) symptom-free leaf at 7 days post-infiltration. **B**) necrosis in leaves 21 days post-infiltration. Note that symptoms occur only around the infiltration site (syringe release) but not in the remaining infiltrated area.

### Protein extraction and analysis

*Arabidopsis thaliana* (Col-O), *Nicotiana* *benthamiana* and *Tropaeolum* *majus* leaves were snap-frozen in liquid nitrogen. Proteins were extracted as previously described [15] separated by SDS-PAGE (12%) and visualised by Coomassie blue staining.

### Additional technical comments

*Tropaeolum* *majus* growth is not seemingly impaired by the presence of agrobacteria, and the plant will form new leaves. An individual plant can be infiltrated multiple times. Transformation efficiency and level of transgene expression of primary and secondary infiltrations (1 week gap) are similar. When all leaves, present at the time of infiltration were used, plants tended to display chlorophyll bleaching and general disease symptoms. As a rule of thumb, a maximum of 70% leaf area can be infiltrated.

## Results

For successful *A. tumefaciens* mediated transformation *via* leaf infiltration two obstacles need to be overcome; one is a “mechanic” issue, the other refers to a plant’s “willingness” or susceptibility to accept foreign DNA from a pathogen. Mechanically, infiltration means the introduction of liquid into leaf tissue. Leaf development involves cell formation that defines the leaf structure, and cell expansion. Rapidly expanding cells have a rather loose cell wall and may therefore be infiltrated more easily. The principal requirements for a candidate plant to offer improvement and/or an alternative to *Nicotiana* , include:

 Easy to grow (space, time, light/humidity)

 Related to *Arabidopsis*

 Multiple leaves of similar width, ideally >5 qcm for easy handling

 Late-flowering, offering a wider time window for infiltrations

 Syringe-introduced liquid should spread readily within the leaf to give large transformation areas but few sites of wounding (point of injection).

*Nicotiana* and *Arabidopsis* belong to the order of Solanales and Brassicales, respectively, and thus are only distantly related (Figure 1). Several members of the Brassicales were critically examined with respect to the above-listed criteria. Nasturtium turned out to be by far the most suitable plant. It belongs to the family of *Tropaeolaceae*, one of the 17 families within the order Brassicales (Figure 1, right).

**Figure 4:**
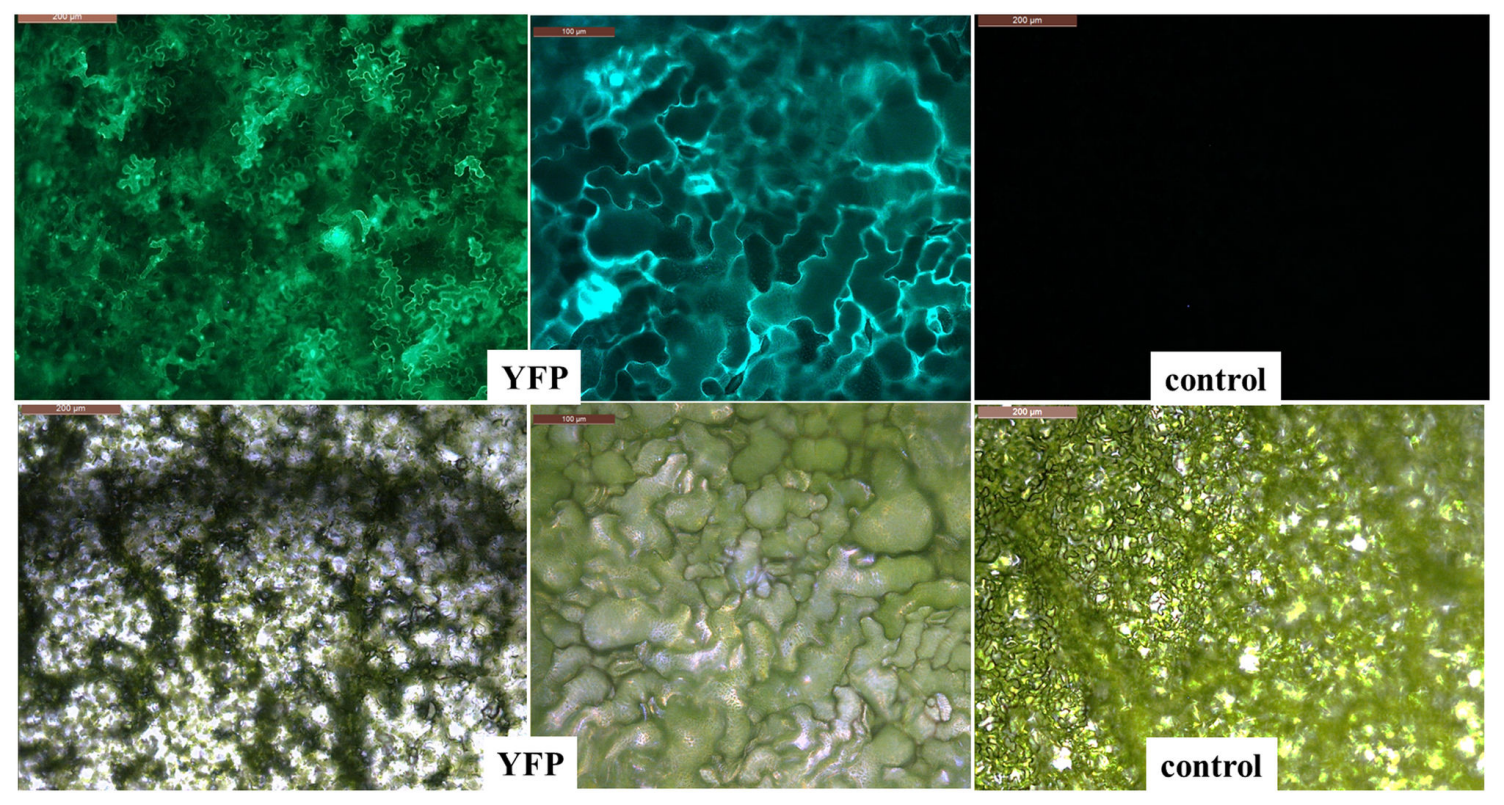
Transient expression of Yellow Fluorescent Protein. *Tropaeolum majus* leaves were infiltrated with agrobacteria carrying a construct for constitutive expression of YFP. Transgene expression was analysed 5 dpi by UV microscopy, using a low (left) and high-magnification objective. Top: fluorescence; bottom: bright-field image. No fluorescence was observed in leaves infiltrated with Agrobacteria carrying a control construct (luciferase) (right). For additional images see figure S1.

**Figure 5:**
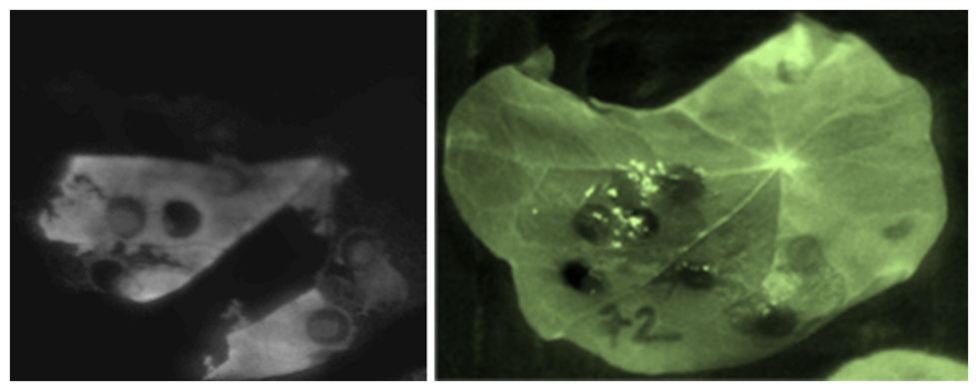
Luciferase activity in transgenic *Tropaeolum* *majus* tissue. *T* *. majus* leaves were infiltrated with agrobacteria carrying a construct for constitutive expression of the luciferase gene. 5 days post-infiltration, an area of the leaf (top left) was infiltrated with substrate solution (10 mM MgCl2, 10 mM MES pH 5.7, 0.5% DMSO, 0.1 mM luciferin), followed by chemiluminescence detection. Left: chemiluminescence (Chemdoc Biorad, exposure 7 sec.). Right: brightfield image of leaves, showing the luciferin-infiltrated area. Note that some areas were not infiltrated with substrate solution only, but not with agrobacteria and *vice versa* to document that the signal is solely due to expression of the reporter transgene.

*Tropaeolum* *majus* seeds were sown directly on soil. Germination was observed within 3 days. Two-week-old plants had formed 4-6 leaves. Plants were tested for their accessibility to agrobacterial infiltration. To this end, a simplified and empirically optimised technique, based on the published transformation procedure of *N* *. benthamiana* [16] was used. An overview of material and steps involved is shown in Figure 2. Infiltration was performed by gently releasing the content of a syringe containing agrobacteria suspension into the abaxial side of leaves. Larger leaves were easier to infiltrate. The uptake of infiltration liquid was detectable as successive spreading of a dark-green zone (Figure 3A). In initial experiments, only single gene transfers were tested: At 5 dpi, leaves transformed with a CaMV35S::YFP construct (for constitutive expression of yellow fluorescent protein) were analysed by UV microscopy. Stunningly, transformation efficiency was >80%, as revealed by the strong fluorescence signal emitted from YFP-expressing cells (Figures 4 and S1). In leaves infiltrated with a control construct (luciferase) there was no detectable fluorescent signal, excluding the possibility that the signal seen in YFP samples derived from autofluorescence. Reciprocally, when leaves were assayed for luciferase activity, only those transformed with CaMV35S::luciferase displayed chemiluminescence upon treatment with the luciferin substrate (Figure 5).

**Figure 6:**
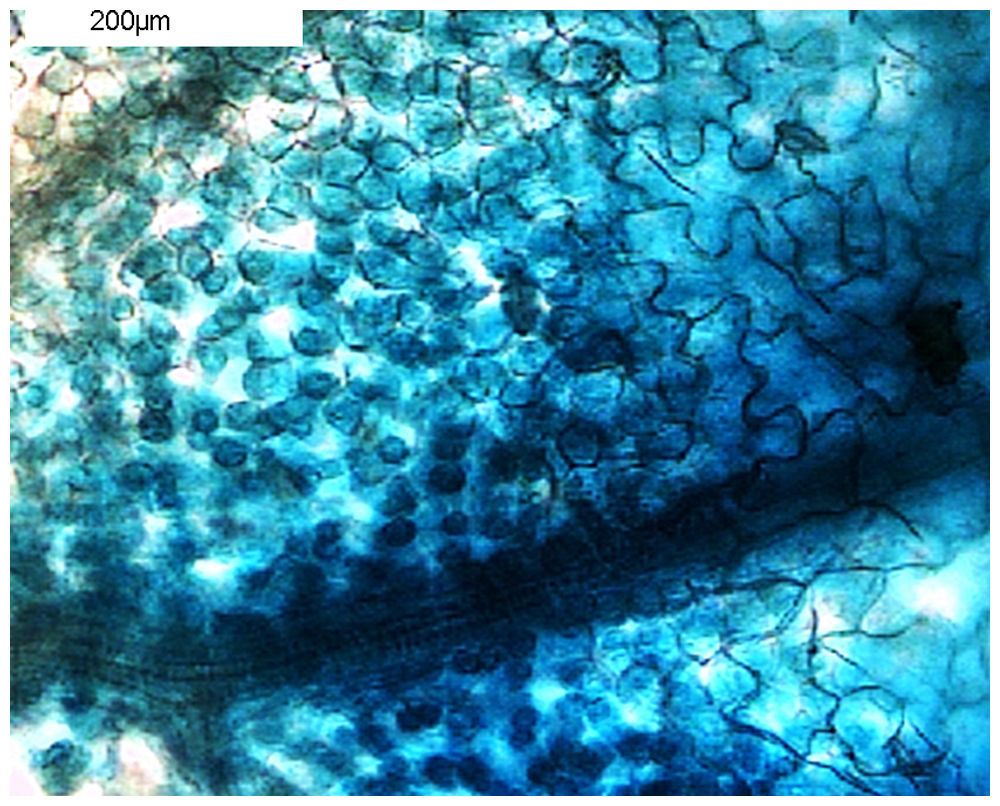
β-glucuronidase activity in transgenic *Tropaeolum* *majus* tissue. Leaves were infiltrated with Agrobacteria carrying a construct for constitutive expression of the GUS reporter gene (including an intron). Enzyme activity was assessed 5 dpi by infiltrating the leaf with substrate solution. The typical blue colour arises from β-glucuronidase-catalysed conversion of the substrate.

**Table 1:** Overview on major features of *Tropaeolum* *majus* agroinfiltration as transient expression system.

**Taxonomic aspects**	*T* *. majus* belongs in the order Brassicales, as does *Arabidopsis* . RNAi-based gene silencing with *Arabidopsis* -derived DNA constructs is more likely to succeed in *Tropaeolum* than in *Nicotiana.*
**Experimental aspects**	**Plant growth**
	Modest space requirements
	Multiple plants grown in the same pot can be bound to the site (long flexible stems).
	Climate-controlled growth cabinet not required
	**Transformation procedure**
	simple and inexpensive
	Expanded leaves are equally suitable, no leaf-to-leaf variation observed.
	Infiltration requires some empirically acquired know-how. The infiltration liquid moves slowly, but gradually (30 sec/8 qcm).
	**Transgene expression and kinetics**
	Transformation efficiency is reproducibly high. > 80% of cells in the infiltrated area express the transgene (here tested for YFP, GUS, Luciferase).
	Expression detectable 4 days-post-infiltration (dpi), observed >10 dpi, (transient, kinetics comparable to *Nicotiana* ).
	Multiple gene transfer possible, e.g. for effector/reporter gene studies; interaction studies (here tested via BiFC assay).
	**Limitations**
	“Lotus effect”: Treatment with liquid effector substances (e.g. elicitors in stress research) may have to be applied by infiltration.
**Perspectives for basic and applied research**	**Relevant tool for basic research, e.g.**
	Endomycorrhizal interactions
	Biosynthesis and metabolism of glucosinolates and oleates
	**Pharma industry and biotechnology**
	May facilitate isolation of key components in biosynthesis of health-promoting substances (for transfer into other species; for *in vitro* synthesis etc.)
	Transgenic *T* *. majus* cell cultures may be established for large-scale production of desired substances and phyto-pharmaceuticals.

To monitor the duration of “transient” expression, YFP-transformed leaves were examined over a 3-week-period. Strong YFP-derived fluorescence was still seen after two weeks, but hardly detectable after three weeks. The appearance of infiltrated leaves indicated that the presence of agrobacteria did not largely affect tissue vitality. At 7 dpi, leaves were virtually symptom-free (Figure 3A). At 21 dpi, necrosis was observed in the narrow area surrounding the infection site (Figure 3B).

Having established the transformation procedure, the suitability of nasturtium leaf infiltration as tool in popular aspects of molecular biology – such as reporter gene activity and protein–protein interaction studies was evaluated. Leaves were infiltrated with agrobacteria carrying a CaMV35S promoter-driven β-glucuronidase (GUS) gene. The GUS gene contains an intron to ensure that GUS activity solely derives from the plant, not from the pathogen. GUS staining in tissue 5 dpi revealed large areas with the typical blue, intensive colour indicating strong GUS activity (Figure 6). Concludingly, nasturtium is compatible with GUS reporter gene activity assays. The intron was recognised and spliced correctly.

**Figure 7:**
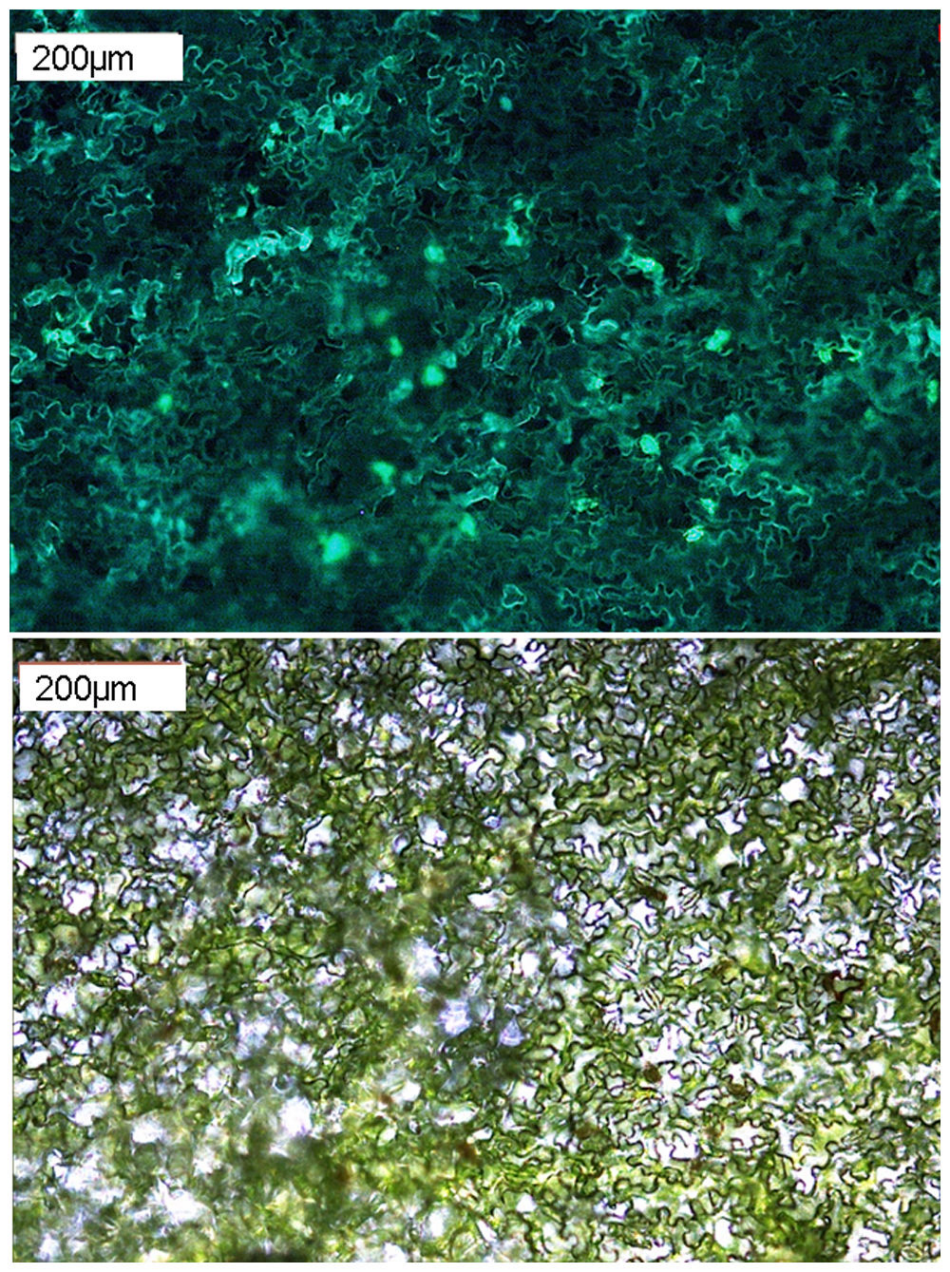
Protein-protein interaction studies in *Tropaeolum majus* tissue. Leaves were infiltrated with Agrobacteria carrying constructs for the constitutive expression of MKK4 and MPK3, fused to the C- and N-terminal fragment of YFP, respectively. Bimolecular fluorescence complementation, documenting the interaction, was detected by UV microscopy, 5 dpi. top: UV image, bottom: bright-field image. For additional images see figure S1.

Next, co-delivery of multiple transgenes for protein–protein interaction studies was tested using the bimolecular fluorescence complementation (BiFC) approach [17]. I chose two well-characterised interacting proteins from *Arabidopsis* , representing components of a mitogen-activated protein kinase cascade [15,18]: MKK4, fused to the C-terminal YFP fragment and MPK3, fused to the N-terminal YFP fragment. UV microscopy revealed positive fluorescence in leaves 5 days after co-infiltration. Signals were observed in cytoplasm and nuclei (Figures 7 and S1), which is in line with the subcellular localisation of MKK4-MPK3 complexes in *Arabidopsis* protoplasts and *Nicotiana* leaves [15].

**Figure 8:**
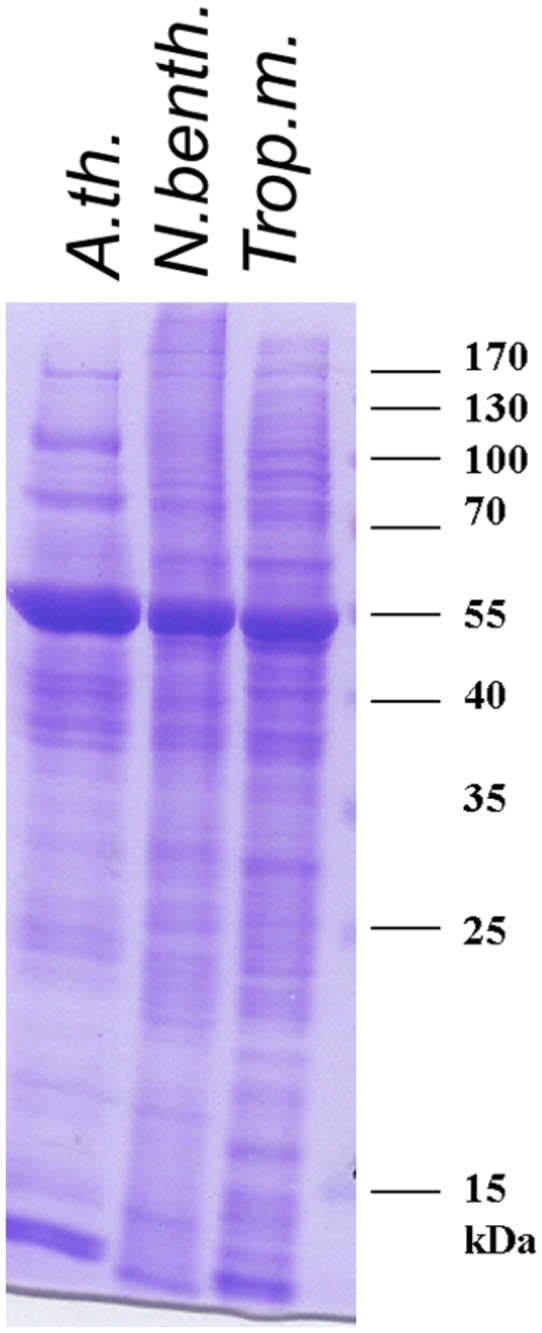
Comparison of protein profiles in *Arabidopsis* *,* *N* *. benthamiana* and *Tropaeolum* *majus* . 20 µg protein extracted from *Arabidopsis* rosette leaves, *Nicotiana* *benthamiana* or *Tropaeolum* *majus* leaves were separated by SDS-PAGE (12%) and visualised by Coomassie Blue staining.

A number of downstream analyses in transient transformation experiments, such as immunoblotting, mass spectrometry and enzymatic assays involve the extraction of proteins. To compare *Arabidopsis* , *N* *. benthamiana* and nasturtium with respect to protein extractability, yield and profile, protein extracts from leaves of these species were prepared and separated by SDS-PAGE. Similar amounts of proteins were obtained (app. 15 µg/mg fresh weight). A dominant, approximately 55 kilo Dalton-sized band was contained in all samples. It most likely represents the large subunit of ribulose bisphosphate carboxylase. Expectedly, there were both similarities and differences in the protein profiles of the three species (Figure 8). As in *Arabidopsis* and *N* *. benthamiana* , nasturtium proteins are stable for at least 7 days (4°C storage and subsequent SDS-PAGE or catalase activity assay, not shown). In summary, functional *T* *. majus* . proteins can be isolated using standard plant protein extraction protocols.

## Discussion and Conclusions

*Tropaeolum* and *Arabidopsis* are phylogenetically related. For unknown reasons, *Arabidopsis* “floral dipping” into suspensions of *A. tumefaciens* is an efficient and well-established method, while leaf infiltration yields poor transformation rates. Plants of the order Brassicales produce characteristic sulfur-rich metabolites, glucosinolates. These mustard-oil glycosides function in the defence against pests and pathogens [19], and might therefore be restrictive factor in *Arabidopsis* leaf transformation. However, in view of the data represented here, glucosinolates are not a barrier for agrobacterial infection. Likewise, this means that other species within the Brassicales may also be accessible to *Agrobacterium* -mediated leaf infiltration. Based on the above-listed characteristics *Tropaeolum* was studied here. Transgene expression in *T* *. majus* was transient, as it was clearly detectable 5-14 days post-infiltration, but not in leaves 3-weeks after infiltration. These expression kinetics are similar to those know from *Nicotiana* . To achieve stable transformation, resistance marker-based selection may be needed. It seems reasonable to assume that whole plants can be generated from transgenic cells via tissue-culturing of surface-sterilised leaves, a question that was not addressed here. I focussed on the advantages of *Tropaeolum* leaf infiltration as transient expression system, documenting its applicability for various experimental approaches in molecular biology. It offers a complimentary tool to established methods and combines some beneficial characteristics of *Arabidopsis* and the established *Nicotiana* transient expression systems (table 1). *Tropaeolum* and *Arabidopsis* genes are functionally replaceable, as had been shown for *FAD2* encoding a fatty acid desaturase [20]. Research fields that may particularly benefit from the here-described expression system include those of xyloglucan metabolism [21], oleate biosynthesis [22] and endosymbiosis [23] and plant-insect interaction [24]. Given the health-promoting effects of *Tropaeolum* *majus* , including utinary tract protection [25,26], antidiuretic [27] and even anti-tumor activity [28], conquering this plant for genetic manipulations harbours potential for biotechnological and pharmacological applications. It may be employed as production platform of tailor-made glucosinolate compounds, similarly to sesquiterpenoid pharmaceutical production in tobacco [11].

## Supporting Information

Figure S1 **Microscopy images supporting the data shown in Figure 4 and Figure 7.** *T* *. majus* leaves were infiltrated with agrobacterial suspensions to induce expression of luciferase (control, left), Yellow fluorescent protein (middle); or for protein–protein interaction studies (MKK4 and MPK3, fused to the C- and N-terminal fragment of YFP, respectively). Transgene expression was analysed 5 dpi by UV microscopy. Top: fluorescence; bottom: bright-field image.
(TIF)
