# Supplementary figures and images for: Tropaeolum Tops Tobacco – Simple and Efficient Transgene Expression in the Order Brassicales
Source: PLoS One. 2013 Sep 10;8(9):e73355. doi: 10.1371/journal.pone.0073355 (PMC3769268; doi:10.1371/journal.pone.0073355)

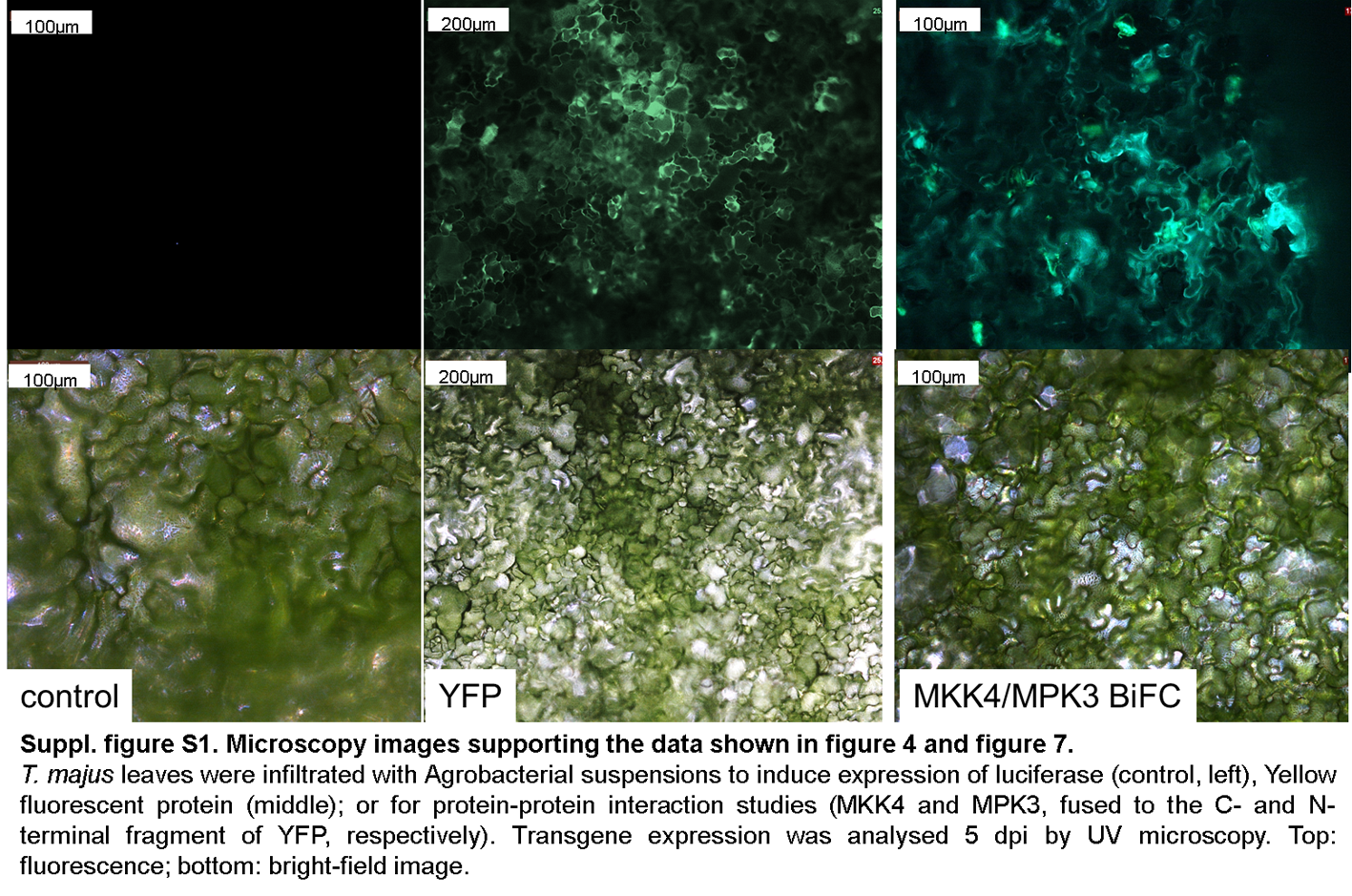

Supplement: Figure S1 — Microscopy images supporting the data shown in Figure 4 and Figure 7. T . majus leaves were infiltrated with agrobacterial suspensions to induce expression of luciferase (control, left), Yellow fluorescent protein (middle); or for protein–protein interaction studies (MKK4 and MPK3, fused to the C- and N-terminal fragment of YFP, respectively). Transgene expression was analysed 5 dpi by UV microscopy. Top: fluorescence; bottom: bright-field image. (TIF) [file pone.0073355.s001.tif]
